# Development and Characterization of Biocompatible Membranes from Natural Chitosan and Gelatin for Pervaporative Separation of Water–Isopropanol Mixture

**DOI:** 10.3390/polym13172868

**Published:** 2021-08-26

**Authors:** Akshay S. Kulkarni, Ashok M. Sajjan, T. M. Yunus Khan, Irfan Anjum Badruddin, Sarfaraz Kamangar, Nagaraj R. Banapurmath, Narasimha H. Ayachit, M. Ashwini, A. Sharanappa

**Affiliations:** 1 Department of Chemistry, KLE Technological University, Hubballi 580031, India; akshaykulkarni@kletech.ac.in; 2 Center for Material Science, KLE Technological University, Hubballi 580031, India; nr_banapurmath@kletech.ac.in (N.R.B.); ayachit@kletech.ac.in (N.H.A.); 3 Research Center for Advanced Materials Science (RCAMS), King Khalid University, Abha 61413, Saudi Arabia; mtatagar@kku.edu.sa (T.M.Y.K.); irfan@kku.edu.sa (I.A.B.); 4 Department of Mechanical Engineering, College of Engineering, King Khalid University, Abha 61421, Saudi Arabia; ssaheb@kku.edu.sa; 5 Department of Food and Industrial Microbiology, University of Agricultural Sciences, Dharwad 580005, India; ashwinim21@gmail.com; 6 Department of Biotechnology, KLE Technological University, Hubballi 580031, India; sharanappaa@kletech.ac.in

**Keywords:** chitosan, gelatin, isopropanol, pervaporation, biocompatibility

## Abstract

Natural polymers have attracted a lot of interest in researchers of late as they are environmentally friendly, biocompatible, and possess excellent characters. Membranes forming natural polymers have provided a whole new dimension to the separation technology. In this work, chitosan-gelatin blend membranes were fabricated using chitosan as the base and varying the amount of gelatin. Transport, mechanical, and surface characteristics of the fabricated membranes were examined in detail by means of the characterizing techniques such as Fourier transform infrared spectroscopy, differential scanning colorimetry, wide angle X-ray diffraction, scanning electron microscope, and thermogravimetric analysis. In order to analyze the water affinity of the developed blend chitosan-gelatin membranes, the percentage degree of swelling was examined. Out of the fabricated membranes, the membrane loaded with 15 mass% of gelatin exhibited the better pervaporation performance with a pervaporation separation index value of 266 at 30 °C for the solution containing 10% in terms of the mass of water, which is the highest among the contemporary membranes. All the fabricated membranes were stable during the pervaporation experiments, and permeation flux of water for the fabricated membranes was dominant in the overall total permeation flux, signifying that the developed membranes could be chosen for efficient separation of water–isopropanol mixture on a larger scale.

## 1. Introduction

Natural polymers are usually obtained from various sources like an animal (polyhydroxyalkanoates, polylactic acid, polybutylene succinate), bacterial fermentation (chitosan, collagen, chitin), vegetable (alginate, polyisoprene, cellulose-based polymers, starch) [1,2]. These natural polymers are currently used as replacements for synthetic polymers because of their peculiar biodegradable nature. Nowadays, these natural polymers have found applications in various fields like drug delivery, hydrogels, biomedicine, food packaging, water treatment, etc. [3,4,5]. These materials find extensive applications in membrane-based separation-based technologies such as ultra filtrations, micro filtrations, gas separations, and pervaporation. One of the noteworthy applications of these biopolymers is found in wastewater treatment. López-Maldonado et al. have done extensive work on the removal of hazardous metal ions and heavy metals from water using many natural substances and polymers [6,7,8,9].

Among these, pervaporation has identified itself as an important separation technique as it separates several close boiling (azeotropic) liquids, including organic–water and organic–organic liquids [10]. In pervaporation, the separation performance depends on several factors, but one of the most vital factors is the membrane. Intrinsic properties of the Membrane (hydrophilicity, hydrophobicity, free volume) usually decide the separation performance in pervaporation applications. These properties usually depend on the membrane materials. Pervaporation separation of water–organic mixtures requires hydrophilic materials like poly (vinyl alcohol) (PVA), polyvinylidenefluoride (PVDF), etc., which are synthetic polymers [11,12]. However, with these synthetic materials, some natural polymers such as cellulose acetate, sodium alginate, bacterial cellulose, and chitosan are also used as base materials due to their excellent membrane forming ability, hydrophilicity, and eco-friendly nature [13,14,15]. Among these, chitosan is one of the most abundant biopolymers which is produced from the deacetylation of chitin. Along with the hydrophilic nature and membrane forming ability, it has some of the most important properties like biodegradability, non-toxicity, and biocompatibility [16,17,18]. Chitosan also has multi-functionality because of the huge number of polar groups present in its structure. Generally, the membrane materials which exhibit good permeability and selectivity factor usually get hampered. The same is observed in the case of chitosan, as it possesses some drawbacks like excessive swelling. The high content of water in the feed will interact with the polar functional groups present on the chitosan, which results in membrane swelling. This leads to higher permeability and lower selectivity [19]. Further, at a lower concentration of water, the driving force of transport across the membrane becomes very low, which leads to the lower flux, which does not serve our purpose as it affects the pervaporation performance.

In order to overcome these drawbacks, M. Vinu et al. prepared chitosan membranes filled with Al-MOF with the intention of studying the effects of defects and structural crystallinity on the pervaporation performance and observed a higher permeation flux of 458 g/m^2^ h along with the selectivity of 2741 [20]. Ewelina et al. prepared polyamide-6 supported composite chitosan membranes and examined them for pervaporation separation of water/alcohol mixtures and achieved a higher total normalised permeation flux of 0.5 μm kg m^−2^ h^−1^ in comparison with the pristine chitosan membrane [21]. D. Achari et al. fabricated polystyrene sulfonic acid-co-maleic acid (PSSAMA) incorporated chitosan polyelectrolyte membranes and obtained better membrane performance (highest separation selectivity of 5352 with a flux of 4.145 × 10^−2^ kg/m^2^ for water/ter-butanol mixture separation) for different water–organic liquid mixtures separation (water-n-propanol, water-1,4 dioxane, water–isopropanol, water-ter-butanol) in comparison with the pristine chitosan membrane [22].

In order to promote and enhance the properties and membrane performance of chitosan, we tried to blend the chitosan with the natural protein gelatin to incorporate its properties in the fabricated chitosan membranes. The purpose of this work is to develop highly efficient and biocompatible pervaporation membranes for the separation of isopropnaol–water mixtures. Gelatin is produced by partial hydrolysis of collagen and is considered one of the important green polymers. Gelatin contains free amino and carboxylic groups on its skeletal. This increases the perm selectivity of gelatin towards water [23]. Moreover, the flexible chain structure of the gelatin leads to the compact and order chain arrangement in the membrane matrix. Along with these properties, the presence of hydrophilic functional groups on the polymer chain makes it more affinitive towards the water. In this work, we have fabricated membranes using chitosan as the base and varied the content of gelatin. The physical, chemical and morphological characteristics of the developed membranes were characterized using various characterization methods such as FTIR, DSC, WAXD, SEM, and TGA. Further, the fabricated membranes were tested rigorously for the separation of water–isopropanol mixture via pervaporation technique at various temperatures and feed concentrations and discussed thoroughly.

## 2. Materials and Methods

### 2.1. Materials

Reagent grade Chitosan (N degree of deacetylation 75–85%, Mw ≈ 200,000) was purchased from Sigma-Aldrich chemicals, St. Louis, MO, USA. Reagent grade gelatin, ethanoic acid, and isopropanol (IPA) were procured from S. D. fine Chemicals Ltd., Mumbai, India. Double distilled deionized water was used in the analysis.

### 2.2. Membrane Fabrication

A fixed weight (3 g) of chitosan is mixed in 100 cc of 2 weight % of an aqueous solution of ethanoic acid and stirred for 24 h at ambient temperature. The prepared homogenous solution is then filtered to get rid of the undissolved particles; the casting procedure was carried out in the room, which was maintained clean and dust-free. The prepared solution was poured uniformly on the glass plate and spread evenly with the help of a casting knife. The casting plate was then left for drying for two days at room temperature. Subsequently, the membrane was peeled carefully, and the obtained pristine membrane was named M-0.

**Figure 1:**
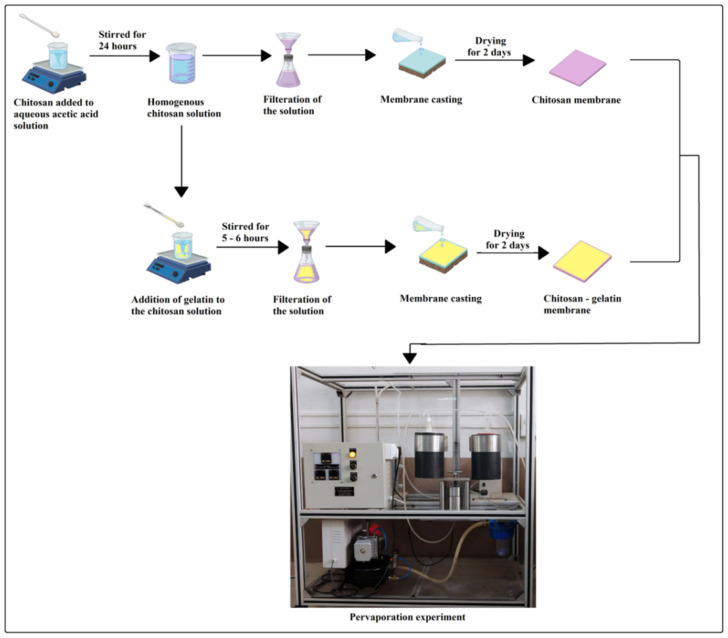
Schematic pathway of the experimental work.

In order to prepare chitosan-gelatin membranes, 5, 10, 15, and 20 % mass of gelatin with respect to the weight of chitosan was added to the previously prepared homogenous chitosan solution. The mixture was then stirred for 5–6 h at 50–60 °C temperature. Then the obtained solution was cast, and the membranes were obtained by following the same procedure explained above. The obtained membranes were then named M-5, M-10, M-15, and M-20, respectively, based on the mass% of gelatin present in the matrix of the membrane. The pathway of the membrane fabrication is illustrated in Figure 1.

### 2.3. Membrane Characterization

In order to understand the chemical interaction between the chitosan and gelatin, FTIR analysis was done using a spectrometer named Nicolet, Impact - 410, Madison, WI, USA. Fabricated membranes were properly ground with the applied pressure of 400 kg/cm^2^_,_ and pellets were prepared using KBr. The spectrum was reported in the spectral range of 400 to 4000 cm^−1^. The influence of the gelatin on the solid-state structure of the membrane was analyzed using Philips Analytical X-ray Diffractometer. The range of the analysis was 5° to 50° at the controlled speed of 8°/min. The glass transition temperature (Tg) and thermal behavior of the fabricated membranes are analyzed using thermogravimetric analysis and differential scanning calorimetry techniques by means of DSC Q 20, TA Instruments, Waters LLC, New Castle, DE, USA. The analysis was done in the presence of N_2_ at the rate of heating of 10 °C/min. With the intention of analyzing the miscibility of the chemicals and surface morphology, scanning electron microscopic images were taken with JSM-400 Å, Tokyo, Japan. Before the analysis, the properly dried membranes were coated with a sputtered gold layer of the thickness of 400 Å.

### 2.4. Equilibrium Swelling

Sorption measurements of the fabricated membranes were analyzed at room temperature. Previously weighed (**W_d_**) membranes samples were dipped in the water–isopropanol test solutions having different compositions in an airtight container. The membranes were allowed to soak in the liquid for 48 h. Then the membranes were taken out and wiped with soft tissue paper in order to remove the surface adhered liquid droplets, and then the weight of the wet membranes (**W_s_**) was analyzed. Using the weights, (**W_d_** and **W_s_**), the percentage degree of swelling was measured using the following expression:

(1) $$DS\;\left(\%\right)=\left[\frac{W_{s}-W_{d}}{W_{d}}\right]\;\times \;100$$

### 2.5. Pervaporation Experiments

**Figure 2:**
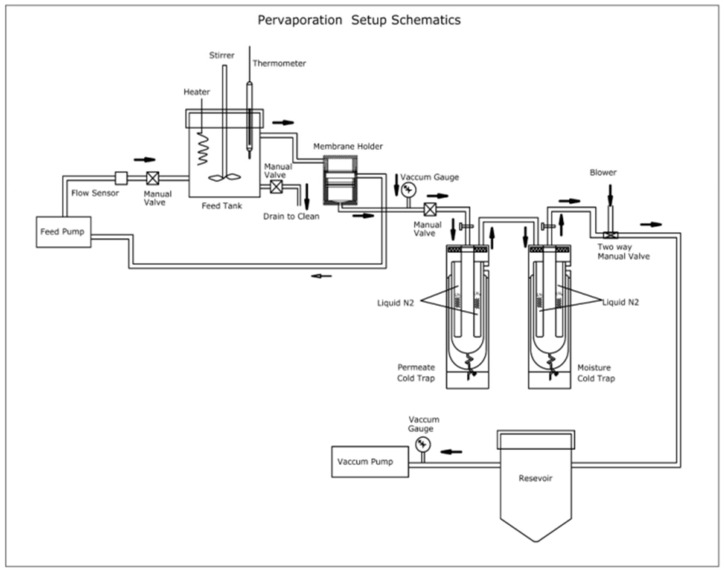
Schematic representation of the pervaporation unit.

The pervaporation experiments were conducted in a specially designed pervaporation unit described in our previous research article [8]. The schematic representation of the pervaporation unit is shown in Figure 2. The unit has a feed tank with a volume capacity of 4 L completely sealed from the surrounding inside a heating jacket. The feed tank is also equipped with a stirrer for uniform maintenance of the temperature of the solution mixture, and a circulating pump is also equipped for the continuous circulation of the solution. The heating jacket consists of a heater with 2.5 KW power rating and a temperature sensor Pt-100 for continuous and uniform heat maintenance in the tank. A highly sophisticated TC513BX auto-tuning temperature controller was installed for utility purposes. Membrane holder having a surface area of 15 cm^2^ is fabricated from high-quality steel, which is chemically inert. The membrane must be installed in the membrane holder and left for 1 h for equilibrium establishment. Then the vacuum is applied from the permeate side. Permeate vapors are then condensed and collected at uniform time intervals. Further, the mass and the composition of the collected sample of the permeate are determined using the digital microbalance and Karl Fischer titrator named KAFI smart.

In order to achieve higher accuracy of the results, experiments were done three times, and the average data are considered for calculations. Using the data, the efficiency of the fabricated membranes for the water–isopropanol separation using pervaporation technique was analyzed by the calculation of separation selectivity (*α_sep_*), total permeation flux *(J*) and pervaporation separation index (*PSI*) using the following equations [24].

(2) $$J=\frac{W}{At}$$

(3) $$\alpha_{sep}=\frac{P_{w}/P_{IPA}}{F_{w}/F_{IPA}}$$

(4) $$PSI=J(\alpha_{sep}-1)$$

where *W* = mass of permeate (kg), *A* = effective membrane area (cm^2^), *t* = permeation time (h), *P_w_* = mass percentage of water in the permeate and *P_IPA_* = mass percentages of IPA in the permeate. *F_w_* = mass percentage of water in the feed and *F_IPA_* = mass percentage of IPA in the feed.

Additionally, the permeance (Pi/l) was calculated using the following equation in order to get an unambiguous idea about the inherent properties of fabricated membranes.

(5) $$\frac{P_{i}}{l}=\frac{D_{i}K_{i}}{l}=\frac{j_{i}}{P_{i}^{f}-P_{i}^{p}}$$

where D_i_ = diffusion coefficient of the i^th^ component, K_i_ = sorption coefficient of the i^th^ component, P_i_ = permeability of the i^th^ component, $P_{i}^{f}$ = feed vapor pressures of the i^th^ component, $P_{i}^{P}$ = permeate vapor pressures of the i^th^ component and l = membrane thickness. j_i_ = molar flux of i^th^ component.

## 3. Results

### 3.1. Membrane Characterization

#### 3.1.1. FTIR Studies

**Figure 3:**
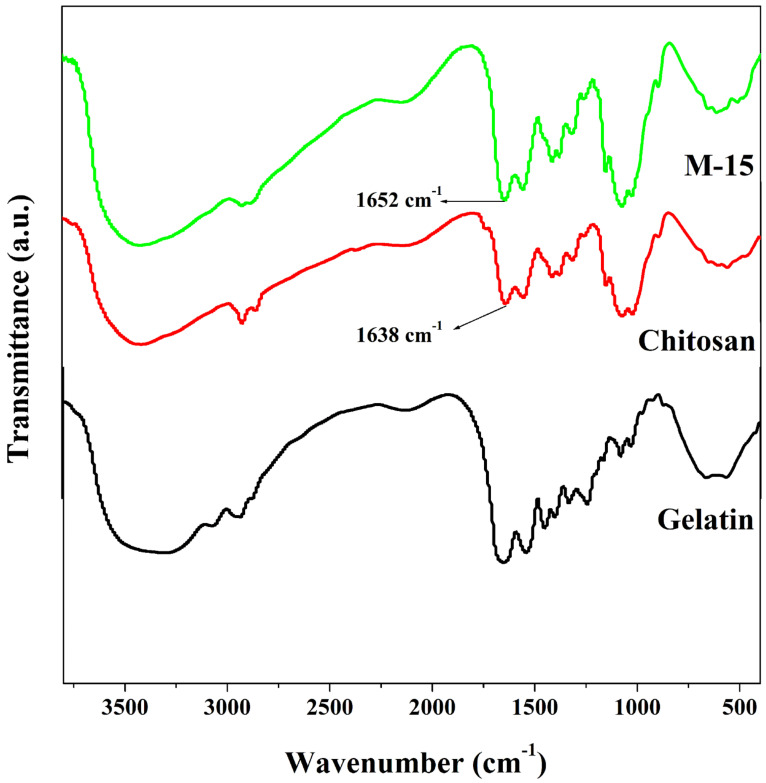
FTIR spectra of plane gelatin, plane chitosan, and chitosan-15 mass% gelatin blend membrane.

In order to understand the molecular fingerprinting of the fabricated membranes, the FTIR analysis was carried out, and the obtained spectra of gelatin, chitosan, and gelatin incorporated chitosan membrane are demonstrated in Figure 3.

**Figure 4:**
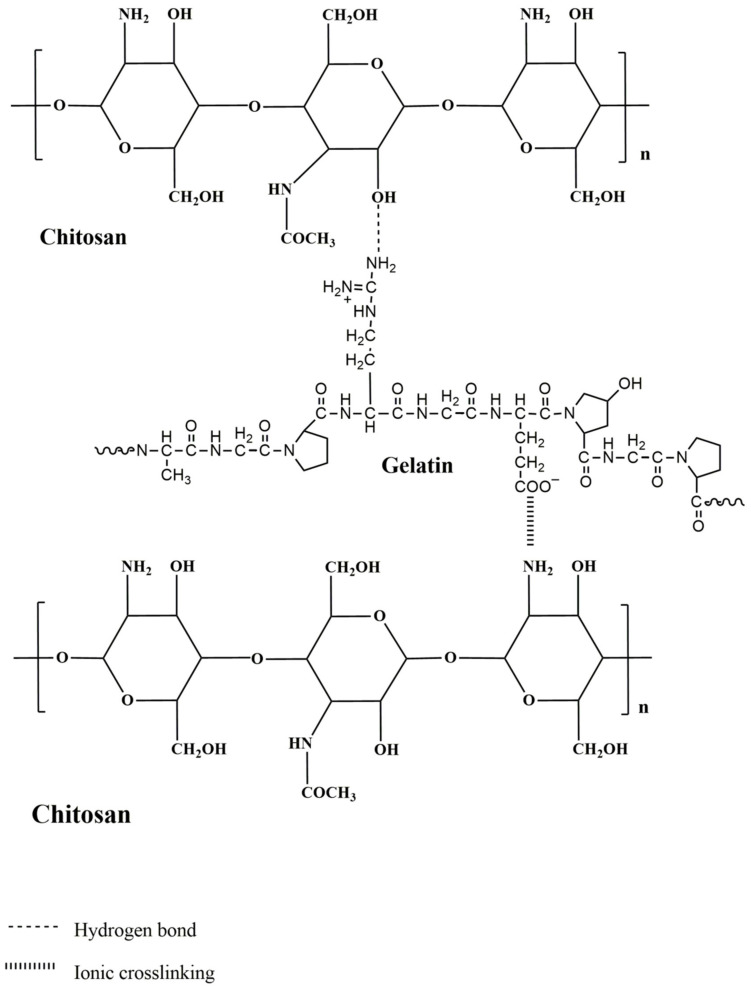
Interactions between chitosan and gelatin.

In FTIR spectrum of gelatin, a broad peak observed around 3410 cm^−1^ is assigned to the stretching N-H group. The -CH stretching vibration peak has appeared at 2931 cm^−1^. Peak visible at 1656 cm^−1^ is assigned to the occurrence of C=O and C-N stretching of amide I. The peaks 1544 cm^−1^ and 1238 cm^−1^ represent N-H bending and C-N stretching of amide I and amide II, respectively [25]. Further in the spectrum of chitosan, the peak at 1152 cm^−1^ corresponds to polysaccharides of chitosan. Peak assigned to N-H and C-N vibrations of amide III is exhibited at 1252 cm^−1^. Peaks at 1560 cm^−1^ and 1638 cm^−1^ are assigned to N-H and C-N group vibrations of amide II and N-H and C=O group vibrations of amide I, respectively. The –CH stretching vibration peak appeared at 2931 cm^−1^. The broadband between the ranges 3400 cm^−1^ to 3500 cm^−1^ was due to N-H stretching and O-H stretching vibrations [26]. However, for chitosan–gelatin membrane, the peaks of amide I for chitosan at 1638 cm^−1^ were shifted to 1652 cm^−1^ along with the increment in the intensity. This can be attributed to the ionic cross-linking between the -COO^−^ of gelatin and -NH^3+^ of chitosan in these composites [27]. The band for O-H stretching vibration present between 3400 cm^−1^ to 3500 cm^−1^ was also seen broadened, which indicates the enhanced inter and intramolecular hydrogen bonding between the two chemicals. This spectral evidence confirms the miscibility of gelatin in the chitosan matrix and the compatibility between the two chemicals. The interactions between chitosan and gelatin are illustrated in Figure 4.

#### 3.1.2. Wide-Angle X-ray Diffraction Studies (WAXD)

**Figure 5:**
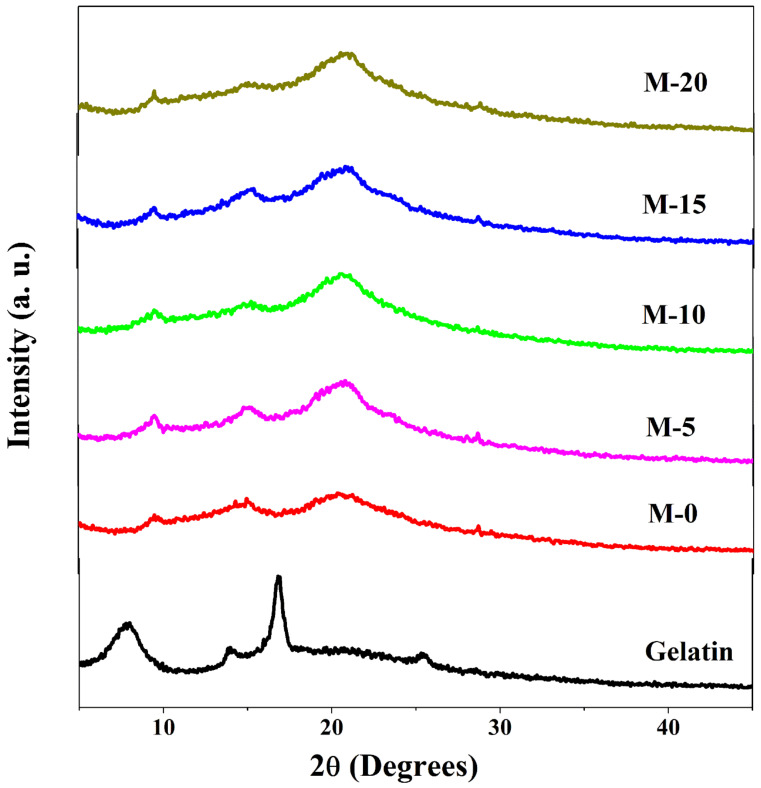
WAXD patterns of gelatin, chitosan, and gelatin incorporated chitosan membranes: pure chitosan; (M); (M-5) 5 mass%; (M-10) 10 mass%; (M-15) 15 mass%; (M-20) 20 mass% of gelatin.

The WAXD studies of the plane gelatin were carried out, and the obtained diffractograms are shown in Figure 5. From the diffractogram, it can be seen that gelatin shows two diffraction peaks at 8° and 18°, respectively [28]. The peak that appeared at 8° corresponds to the diameter of the double helix, and the intensity of that peak is correlated to the triple helix segments of the gelatin. The peak appearing at 18° is characterized by the semi-crystalline nature of the gelatin [23]. In order to analyze the intrinsic nature, the fabricated membranes were tested for WAXD studies, and obtained results are also represented in Figure 5. Plane chitosan membrane (M-0) demonstrated a couple of small and sharp peaks at around 2ϴ = 10° and 15° along with a broad peak at around 2ϴ = 21°. The broad peak represents the amorphous part, and the small sharp peaks signify the semi-crystalline part of the membranes [29]. The degree of crystallinity for M-0 was 69%, which is almost in accordance with the literature [30]. In the curves of chitosan–gelatin hybrid membranes, the peaks observed prominently in gelatin are absent, which indicates the chemical interaction and compatibility between the two chemicals. After incorporating gelatin in the chitosan matrix, the intensity of the peak appeared at 2ϴ = 21° gradually decreased along with the increase in the mass% of gelatin up to 10 mass%. This occurs for the reason that at lower concentrations of gelatin, chitosan dominates the complex triple helix structure of the gelatin by the establishment of intermolecular interactions such as ionic cross-linking and hydrogen bonding. This leads to a decrease in the crystalline nature of the membranes (Degree of crystallinity of M-10 = 64%). However, when the concentration of the gelatin increases by more than 10 mass% in the chitosan matrix, the dominant character of chitosan is held back, which leads to the enhancement of the intensity in the diffraction peaks. This leads to a further increase in the crystalline nature of the membranes (Degree of crystallinity of M-20 = 76%).

#### 3.1.3. Thermogravimetric Analysis (TGA)

**Figure 6:**
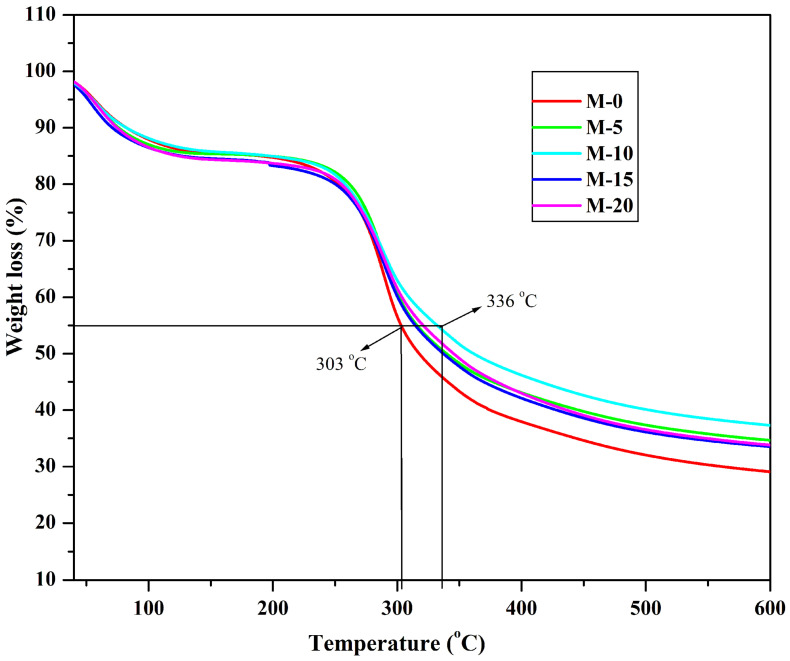
TGA curves of chitosan and gelatin incorporated chitosan membranes: pure chitosan; (M); (M-5) 5 mass%; (M-10) 10 mass%; (M-15) 15 mass%; (M-20) 20 mass% of gelatin.

TGA is an important characterizing technique to analyse the thermal stability of the developed membranes [31]. In order to analyze the degradation behavior and thermal solidity of the developed membranes, thermogravimetric analysis was carried out and the thermograms obtained are illustrated in Figure 6. From the results, it was clearly observed that the first degradation process takes place owing to the loss of water molecules (physically absorbed) from the ambient temperature and 126 °C [32]. These molecules of water exist in the bound state but not in free form. The loss of weight due to the removal of water molecules is about 12 to 15%. On careful observation, we found that that the weight loss was more in the case of M-15 and M-20 compared to the other membranes, and M-10 experienced the least weight loss, which indicates the M-15 and M-20 have higher water affinity, and M-10 has the least water affinity among the contemporary membranes. The decomposition of the second stage started from 225 °C in which the major amount of weight loss is observed owing to the polymeric chains decomposition. If we consider 45% weight loss as a reference point, M-0 experiences 45% weight loss at the lower temperature of 303 °C, which is at the lower temperature compared to the other membranes, whereas M-10 experiences the same weight loss at 336 °C. This illustrates the higher thermal stability of M-10 compared to the other membrane due to the higher ionic interactions and hydrogen bonding between chitosan and gelatin.

#### 3.1.4. Differential Scanning Calorimetry (DSC)

**Figure 7:**
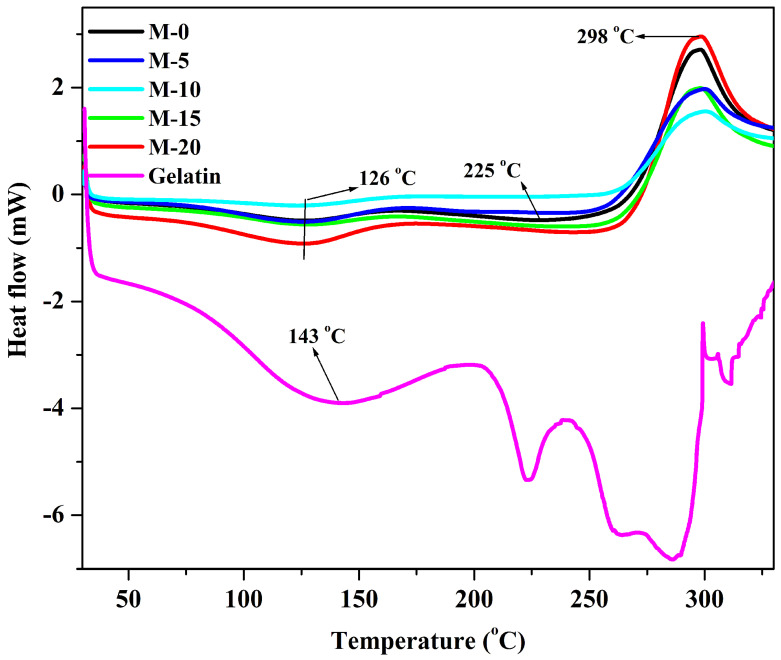
DSC thermograms of gelatin, chitosan and gelatin incorporated chitosan membranes: pure chitosan; (M); (M-5) 5 mass%; (M-10) 10 mass%; (M-15) 15 mass%; (M-20) 20 mass% of gelatin.

The DSC thermograms of plane gelatin, chitosan, and gelatin are illustrated in Figure 7. From the figure, it is noticed that the plane gelatin exhibits an endothermic peak at around 143 °C which may be due to the gelatin denaturation. Further, the intense peak (endothermic) observed at around 288 °C indicates the change of the phase of gelatin as at 320 °C, the melting point of the gelatin is observed [33]. The DSC thermograms of the prepared membranes are also illustrated in Figure 7. In plane chitosan membrane (M), the first endothermic peak appears at around 126 °C, which corresponds to the evaporation of the water molecules (physically absorbed) attached to the internal chains [34]. On careful observation, we see that the area under the curve for M-15 and M-20 is larger compared to the other membranes, which signifies the higher water affinity of the membranes M-15 and M-20. The next peak was observed at around 225 °C with an onset of 175–180 °C, which may be the glass transition temperature of the developed membranes. Here, the weight loss is observed due to the breakage of ionic interactions between the carboxylic group of gelatin and amino groups of chitosan and inter and intramolecular hydrogen bonding between the amino, hydroxyl, and carbonyl groups of chitosan and gelatin. Therefore, for M-0, the decomposition is at a lower temperature compared to the gelatin incorporated chitosan membranes as there are no ionic interactions. However, there is still uncertainty regarding the glass transition temperature as there are different Tg values discussed in the literature [35].

This is owing to the discrepancies in the nature of chitosan, such as deacetylation degree and molecular weight. Further, an exothermic peak is observed at around 298 °C, which is due to the depolymerization or decomposition of biopolymers (glycoside bond cleavage, decomposition of the diacetyl and acetyl units, and monomer dehydration). These results are in accordance with the results of TGA for the thermal stability of the developed membranes.

#### 3.1.5. Scanning Electron Microscopy (SEM)

**Figure 8:**
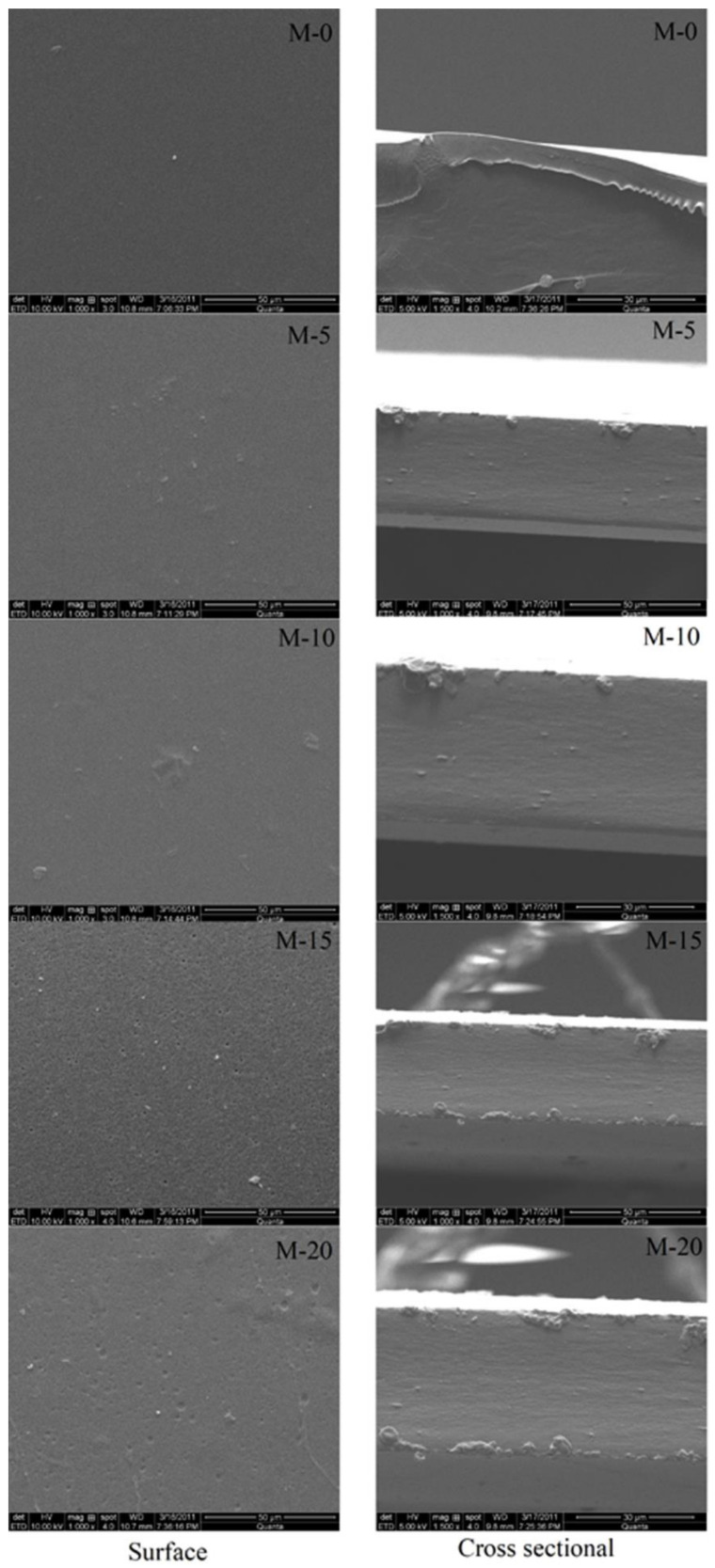
Surface and cross-sectional SEM images of chitosan and gelatin incorporated chitosan membranes: pure chitosan; (M); (M-5) 5 mass%; (M-10) 10 mass%; (M-15) 15 mass%; (M-20) 20 mass% of gelatin.

In order to analyze the surface characteristics of the developed membranes, SEM analysis of the developed membranes was carried out, and resultant SEM images are presented in Figure 8. From the surface, it was clearly noticed that the membrane surfaces are even and homogenous up to M-10. However, when the gelatin content is enhanced by more than 10 mass %, the surface becomes rough and irregular with the presence of few small cavities. This indicates that the compatibility between chitosan and gelatin is high up to 10 mass% of gelatin. However, when we observed the cross-sectional views of the membranes, no roughness or porosity is observed. These results are in agreement with the DSC, TGA, and XRD results. Above 10 mass% of gelatin, the compatibility between chitosan and gelatin is slightly reduced as the gelatin just entangles on the surface of the chitosan.

### 3.2. Effects of the Amount of Gelatin and Feed Composition on Membrane Swelling

**Figure 9:**
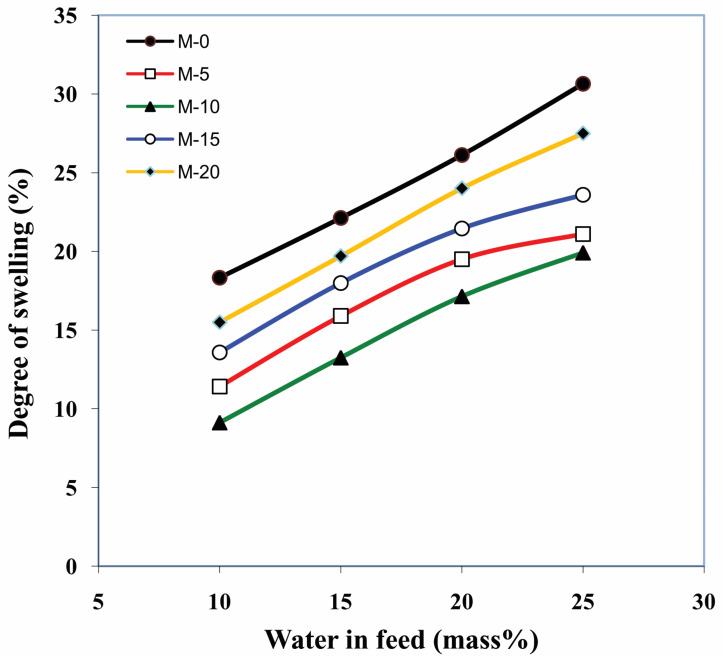
Effect of gelatin and water content on membrane sorption.

Membrane sorption is an important factor for the membranes as it describes the nature of the membranes [36]. With the purpose of assessing the effect of gelatin content and mixture composition on the wettability of the membranes, the % degree of sorption for the developed membranes at various feed concentrations at 30 °C was plotted in Figure 9. From the plots, it was observed that the sorption % was consistently increased as the content of water increased in the mixture. This behavior was obvious because of the strong interaction that exists between the membrane surface and the water molecules. Water molecules, having higher polarity than isopropanol, interact with the polar groups like carbonyl, hydroxyl, amino and carboxylic groups of the membrane surface. However, the sorption % was decreased from the membrane M-0 to M-10. This is mainly because of the ionic cross-linking between the carboxylic group of gelatin and amino groups of chitosan and inter and intramolecular hydrogen bonding between the carbonyl, hydroxyl, and amino groups chitosan and gelatin, which leads to the stiffness in the complex polymer chains. This also leads to a decrease in the degree of crystallization (64% for M-10). Further, as we move to membranes M-15 and M-20, the % sorption was enhanced. This behavior was observed because the gelatin added more than 10 % in the membrane matrix could not form ionic interactions and hydrogen bonding and instead just remained entangled with the chitosan in the matrix. This brings about an increment in the degree of crystallization (76% for M-20). As a result of this, more and more number of polar groups in the membrane matrix will be accessible for the polar interaction with the water molecules (solvent). These results are in good agreement with the DSC, TGA, and WAXD results.

### 3.3. Effects of the Amount of Gelatin and Feed Composition on Pervaporation

The effect of the concentration of water in the separation mixture on the total permeation flux (*J*) and separation selectivity (*α_sep_*) was analyzed at 30 °C. The obtained results are depicted in Table 1.

From the results observed, it is clear that as the water content is increased in the mixture to be separated, the permeation flux for all the membranes increased almost linearly. This is owing to the selective interaction of the fabricated membranes with the polar water molecules. In contrast, the total permeation flux was declined from M to M-10. A similar trend is observed in the case of permeance (Table 2).

This is attributed to the ionic interactions between the carboxylic group of gelatin and amino groups of chitosan and inter and intramolecular hydrogen bonding between the amino, hydroxyl, and carbonyl groups of chitosan and gelatin, which leads to the considerable decrease in the polymer chain mobility and the free volume. Moreover, the unavailability of polar groups for the solvent interaction makes the membranes less permeating. However, for the M-15 and M-20, the permeation flux was enhanced substantially throughout the entire range of compositions in the feed. This is expected as the gelatin added beyond 10 mass% in the membrane matrix could not form ionic interactions and hydrogen bonding and instead just remained entangled with the chitosan in the matrix. This makes the gelatin much more free and relaxed, and more interactive groups like -OH^−^ and -NH^3+^ are available for solvent interactions.

**Figure 10:**
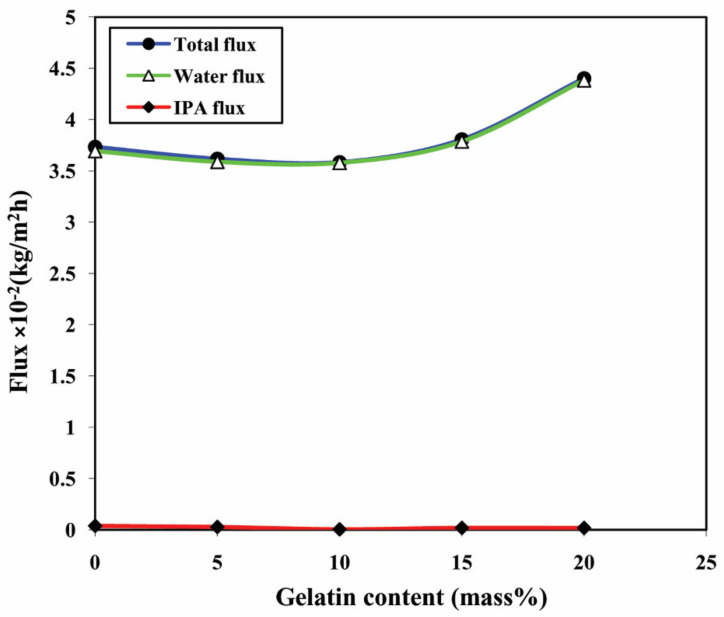
Effect of the content of gelatin on the total permeation flux, permeation flux of water, and permeation flux of isopropanol.

To facilitate the extent of permeation flux for water and isopropanol individually, the Permeation flux of water and isopropanol are calculated individually at 10% aqueous isopropanol mixture and illustrated in Table 3. From the values, it is seen that the permeation flux of water is roughly the same as that of overall permeation flux, and comparatively, the permeation flux of IPA is very small. A similar trend is also seen from Figure 10, which represents overall permeation flux and the individual isopropanol and water permeation fluxes regarded as the function of gelatin content in the membrane for the 10% aqueous isopropanol mixture. These trends clearly depict the selective nature of the developed membranes towards the polar component in the mixture (water).

Selectivity of the membrane commonly relies on the interaction of the membrane with the diffusing molecules and the molecular size of the diffusing molecules. From Table 1, it is clearly seen that the separation selectivity of all the developed membranes declined as the ratio of water is enhanced in the separating mixture. This is caused by the higher interaction taking place between the fabricated membranes and the water molecules.

**Figure 11:**
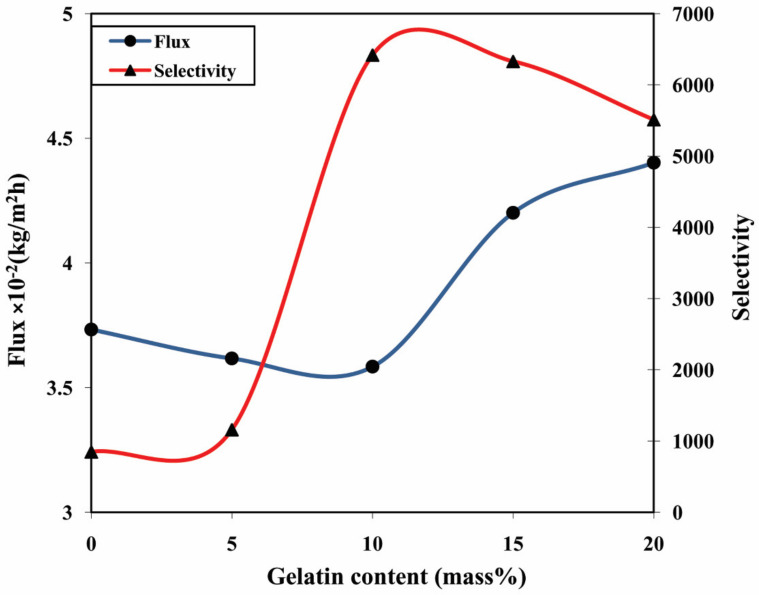
Effect of gelatin on the total permeation flux and separation selectivity of the fabricated membranes.

In contrast, the separation selectivity was increasing from M to M-10 for all the compositions of feed. Mainly this occurs because of two factors. First, this is owing to the interionic interactions and hydrogen bonding between the gelatin and the chitosan, which causes a considerable decrease in the polymer chain mobility and the free volume. Secondly, it is due to the difference in the sizes of the two molecules in the mixture. The size of the isopropanol is comparatively larger than water molecules. Further, for M-15 and M-20, the separation selectivity declined radically. This occurs due to the gelatin added beyond 10 mass% in the membrane matrix not being able to form ionic interactions and hydrogen bonding and instead just remaining entangled with the chitosan in the matrix. Consequently, the gelatin augmented the interaction with the liquids under separation with no decrease in the free volume of the membrane, which led to the decrement of separation selectivity. Figure 11 depicts the plots of total permeation flux plus the separation selectivity plotted with respect to various contents of gelatin in the membrane for 10% aqueous isopropanol mixture separation.

### 3.4. Effect of Gelatin on Pervaporation Separation Index (PSI)

**Figure 12:**
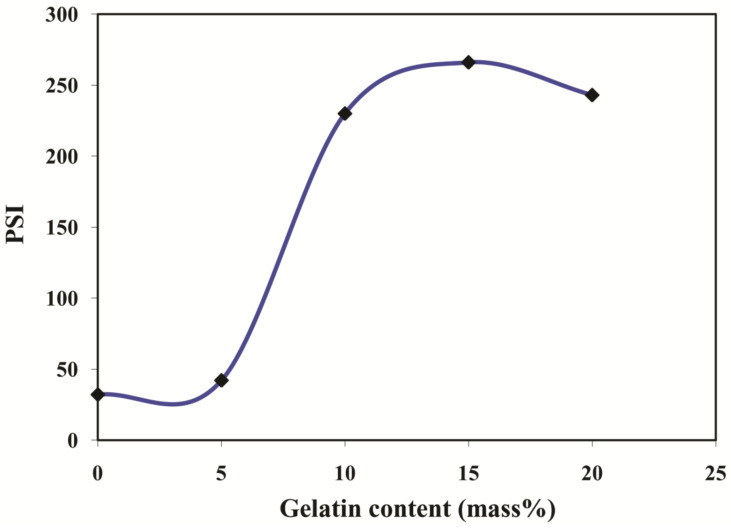
Effect gelatin content on the PSI at 10 mass% of water in the feed.

PSI is the parameter that considers both separation selectivity and total permeation flux into account and depicts the overall membrane performance in pervaporation. This parameter can be regarded as a tool to design novel membranes and to select the optimal membrane for an overall better PV performance. Figure 12 demonstrates the plot of PSI at various content of gelatin for 10% aqueous isopropanol mixture separation. The plot shows that M-15 exhibits the maximum PSI in comparison with its contemporary membranes as it possesses excellent separation selectivity and decent total permeation flux. This proves that the M-15 has better separation ability compared to the contemporary membranes.

### 3.5. Effect of Temperature on Membrane Performance

The influence of temperature during the separation on the membrane performance for 10% aqueous isopropanol mixture separation is analyzed, and the obtained result is depicted in Table 4. From the values, it is seen that the total permeation flux augmented systematically as the temperature increased from 30 °C to 50 °C. Conversely, the separation selectivity declined. This behavior is obvious as higher temperatures will decrease the intermolecular interaction between the membrane and the permeating molecules; in addition, they also decrease the intermolecular interaction in the membrane materials, which leads to the membranes showing plasticizing effect owing to excessive swelling. As a result, the diffusion of associated molecules along with the diffusion molecules turns out to be simple, leading to an enhanced total permeation flux and suppressed selectivity.

## 4. Conclusions

In this work, chitosan–gelatin blend membranes were fabricated using chitosan as the base and varying the amount of gelatin using the conventional casting technique. After the fabrication, the membranes were subjected to the separation of an isopropanol–water mixture using the pervaporation technique at various conditions of temperature and concentration of separating mixtures. In the FTIR, the peak of C=O of amide I for chitosan at 1638 cm^−1^ was shifted to 1652 cm^−1^ along with the increment in the intensity. This confirmed the ionic cross-linking between -NH_3_^+^ of chitosan and the -COO^−^ of gelatin in three composites. From WAXD, it is confirmed that the crystalline nature of the membrane decreases until the 10 mass% of gelatin and is further enhanced by 15 and 20 mass% of gelatin in the matrix. From the DSC and TGA, it is confirmed that the M-10 membrane exhibited the highest thermal stability among the contemporary membranes. Further, from the SEM analysis, it is clear that the compatibility between chitosan and gelatin is good in all the fabricated membranes. In pervaporation analysis, all the membranes with various content of gelatin in the membrane showed good separation selectivity and decent water permeation flux owing to the considerable improvement in the hydrophilicity and the amorphous nature. Out of the fabricated membranes, the membrane loaded with 15 mass% of gelatin (M-15) exhibited the better pervaporation performance with a pervaporation separation index value of 266 at 30 °C for the solution containing 10% in terms of the mass of water, which is the highest among the contemporary membranes.

## Figures and Tables

**Table 1:** Permeation flux and separation selectivity of all the membranes for different mass% of water in the feed at 30 °C.

Mass% of Water	*J* × 10^−2^ (kg/m^2^ h)					*α_sep._*				
	M-0	M-5	M-10	M-15	M-20	M-0	M-5	M-10	M-15	M-20
10	3.733	3.617	3.584	4.201	4.402	848.1	1160	6420	6330	5512
15	6.428	6.350	6.138	6.355	6.605	730.3	879.8	2018	2002	1604
20	9.701	9.076	8.776	9.012	10.21	201.1	412.7	621.0	618.0	405.5
25	14.50	14.01	12.09	12.95	14.59	151.0	174.5	197.0	195.6	134.6

**Table 2:** Permeance for all the membranes for different mass% of water in the feed at 30 °C.

Mass% of Water	Pi/l(GPU) *				
	M-0	M-5	M-10	M-15	M-20
10	829	803	796	933	978
15	1428	1411	1364	1412	1467
20	2156	2017	1950	2002	2269
25	3222	3113	2687	2877	3242

* GPU = gaspermeationunit = 10^−6^ cc(STP)/cm^2^/s/cm Hg.

**Table 3:** Permeation flux of water and isopropanol for all the membranes for different mass% of water in the feed at 30 °C.

Mass% of Water	*J* w × 10^−2^ (kg/m^2^ h)					*J* IPA × 10^−2^ (kg/m^2^ h)				
	M-0	M-5	M-10	M-15	M-20	M-0	M-5	M-10	M-15	M-20
10	3.694	3.589	3.579	4.194	4.395	0.039	0.028	0.005	0.007	0.008
15	6.378	6.309	6.121	6.337	6.580	0.049	0.041	0.017	0.018	0.024
20	9.512	8.989	8.720	8.954	10.11	0.189	0.087	0.056	0.058	0.100
25	14.14	13.77	11.91	12.77	14.27	0.362	0.236	0.181	0.183	0.318

**Table 4:** Permeation flux and separation selectivity of all the membranes for 10 mass% of water in the feed at various temperatures.

Temperature (°C)	*J* × 10^−2^ (kg/m^2^ h)					*α_sep_.*				
	M-0	M-5	M-10	M-15	M-20	M-0	M-5	M-10	M-15	M-20
30	3.733	3.617	3.584	4.201	4.402	848.1	1160	6420	6330	5512
40	5.241	5.180	5.174	5.364	6.129	523.5	722.7	1791	1397	922.7
50	6.640	6.321	6.299	7.299	9.982	452.5	652.7	1159	928.5	723.5

## Data Availability

The study does not reported any data.
